# Radiomics Detection of Pulmonary Hypertension via Texture-Based Assessments of Cardiac MRI: A Machine-Learning Model Comparison—Cardiac MRI Radiomics in Pulmonary Hypertension

**DOI:** 10.3390/jcm10091921

**Published:** 2021-04-28

**Authors:** Sarv Priya, Tanya Aggarwal, Caitlin Ward, Girish Bathla, Mathews Jacob, Alicia Gerke, Eric A. Hoffman, Prashant Nagpal

**Affiliations:** 1 Department of Radiology, University of Iowa Carver College of Medicine, Iowa City, IA 52242, USA; girish-bathla@uiowa.edu (G.B.); eric-hoffman@uiowa.edu (E.A.H.); prashant-nagpal@uiowa.edu (P.N.); 2 Department of Family Medicine, University of Iowa Carver College of Medicine, Iowa City, IA 52242, USA; tanya-aggarwal@uiowa.edu; 3 Department of Biostatistics, University of Iowa College of Public Health, Iowa City, IA 52242, USA; caitlin-e-ward@uiowa.edu; 4 Department of Electrical Engineering, University of Iowa College of Engineering, Iowa City, IA 52242, USA; mathews-jacob@uiowa.edu; 5 Department of Pulmonary Medicine, University of Iowa Carver College of Medicine, Iowa City, IA 52242, USA; alicia-gerke@uiowa.edu; 6 Roy J. Carver Department of Biomedical Engineering, University of Iowa College of Engineering, Iowa City, IA 52242, USA

**Keywords:** cardiac MRI, radiomics, pulmonary hypertension, machine learning, texture

## Abstract

The role of reliable, non-invasive imaging-based recognition of pulmonary hypertension (PH) remains a diagnostic challenge. The aim of the current pilot radiomics study was to assess the diagnostic performance of cardiac MRI (cMRI)-based texture features to accurately predict PH. The study involved IRB-approved retrospective analysis of cMRIs from 72 patients (42 PH and 30 healthy controls) for the primary analysis. A subgroup analysis was performed including patients from the PH group with left ventricle ejection fraction ≥ 50%. Texture features were generated from mid-left ventricle myocardium using balanced steady-state free precession (bSSFP) cine short-axis imaging. Forty-five different combinations of classifier models and feature selection techniques were evaluated. Model performance was assessed using receiver operating characteristic curves. A multilayer perceptron model fitting using full feature sets was the best classifier model for both the primary analysis (AUC 0.862, accuracy 78%) and the subgroup analysis (AUC 0.918, accuracy 80%). Model performance demonstrated considerable variation between the models (AUC 0.523–0.918) based on the chosen model–feature selection combination. Cardiac MRI-based radiomics recognition of PH using texture features is feasible, even with preserved left ventricular ejection fractions.

## 1. Introduction

Pulmonary hypertension (PH) is a disabling disease with long-term morbidity and mortality. The incidence of PH is increasing, which is associated with substantial health and economic implications [1]. PH is defined as when resting mean arterial pressure as measured during right heart catheterization (RHC) is ≥25 mm Hg [2]; however, it was recently proposed to reduce the threshold to define PH to >20 mm Hg [3]. Patients with PH have non-specific symptoms including dyspnea, chest pain, syncope, palpitations, or reduced exercise tolerance [4]. No clinical, laboratory, or ECG marker is specific for early diagnosis of PH. As such, patients often present late at an advanced stage of disease with features of right heart failure. Evidence suggests that early diagnosis and treatment leads to improvement in the long-term prognosis of PH patients [5]. 

Diagnosis of patients with suspected PH and follow-up of patients with suspected PH remains a challenge. Currently, the RHC is considered the gold standard modality to confirm PH. However, RHC is invasive and associated with a 1% risk of major complications and a 0.05% mortality risk [6]. RHC should be chosen selectively in patients with a high degree of suspicion. Non-invasive imaging modalities such as echocardiography, cardiac MRI (cMRI), or chest CT may also provide evidence of PH and help in detecting changes of PH, and each has its own merits and demerits. Echocardiography is highly operator-dependent and may be unreliable or difficult to perform in patients with poor acoustic windows due to body habitus, or in patients with significant lung disease [7]. Echocardiography also has limited sensitivity and specificity in diagnosing PH compared to RHC [8]. A dilated main pulmonary artery >3 cm is a common CT marker for raising suspicion of PH in patients with high risk (left heart disease, obstructive sleep apnea, chronic kidney disease requiring dialysis, systemic sclerosis, congenital heart disease, or sickle cell disease) [9]. However, pulmonary artery diameter alone is inadequate to diagnose PH [9]. cMRI is also commonly used for functional evaluation once PH is confirmed to assess disease severity, right ventricular volume, and ejection fraction. 

Radiomics is an emerging field in medicine that extracts quantitative features from the routinely acquired images that are not visualized by the human observer. Radiomics can be employed to extract features from medical images based on shape, texture, and intensity. The advantage of a radiomics approach is that it can be applied to clinically-acquired images and requires no additional image acquisitions or changes in protocol. Texture features, which are the essential component of radiomics, have been used extensively in the field of neuro-oncology to differentiate tumors, or to separate pathological subtypes of the same tumor [10,11,12,13,14,15]. A few prior studies have evaluated the impact of radiomics-based texture feature extraction in cMRI for diagnosis of myocarditis and differentiation of various cardiomyopathies [16,17,18,19]. However, to the best of our knowledge, the role of radiomics applied to cMRI texture features in the evaluation of PH has not been studied before, and this is the goal of the study presented here. In this study, we have extracted texture features from cMRI of patients with known PH and compared it with patients without PH to identify a subset of texture features which will provide a predictive model for PH. We provide here an investigation of the impact of multiple feature selection and machine learning models on the overall model performance in diagnosing PH. 

## 2. Materials and Methods

**Figure 1:**
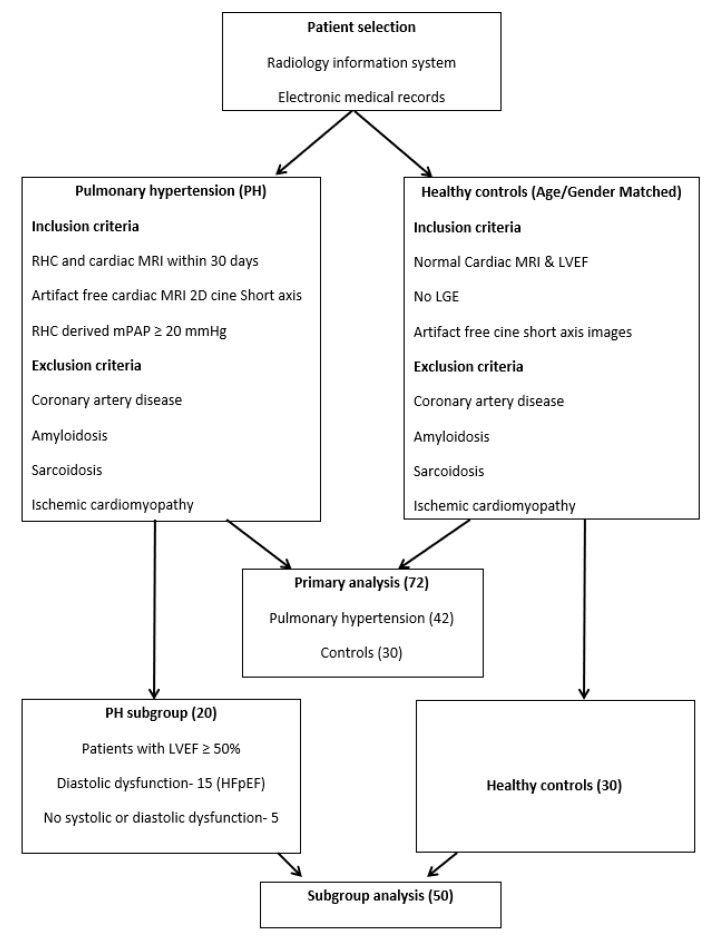
Patient selection. Inclusion criteria for patients with pulmonary hypertension and controls.

This is a single institution retrospective study approved by the local institutional review board. Patients were identified using a radiology information system and electronic medical records. Selection criteria for the PH group was as follows: right heart catheterization (RHC) and cMRI performed within thirty days of either exam, and availability of RHC derived mean pulmonary artery pressure and artifact-free balanced steady-state free precession (bSSFP) 2D short-axis cine cardiac MRI images. Patients with evidence of coronary artery disease, ischemic cardiomyopathy, or suspected infiltrative cardiomyopathy (like sarcoidosis or amyloidosis) on cMRI were then excluded. PH was defined based on RHC derived mean pulmonary artery pressure >20 mm Hg, founded on the recently proposed criteria and clinical classification [3]. Many patients had more than one cause of pulmonary hypertension (for example, lung disease and left heart failure). In these patients, the final class characterization of PH was based on a local multidisciplinary PH team discussion and recommendations. The patients in the PH group included all WHO classes of PH with the majority being from group 2. An age and gender-matched control group was retrospectively selected from patients who had undergone cMRI as part of a clinical work-up for a family history of cardiovascular disease, and a normal cMRI with normal biventricular ejection fraction and no delayed myocardial enhancement. Patients in the control group had no evidence of coronary artery disease, and no history of cardiac interventions including valve replacement or coronary interventions. Patients in the PH and control group were also screened for presence of comorbidities including diabetes mellitus, hypertension, and smoking. This yielded a total of 72 patients, 42 with PH and 30 controls. Primary extraction of texture features derived from cMRI of PH and controls subjects was performed in a consistent way for all the patients in both of the groups. To further analyze the texture features that may help differentiate the control group from PH patients with preserved LVEF, a subgroup analysis was also performed. This subgroup included patients from the PH group who had preserved left ventricle ejection fraction (LVEF) ≥ 50% [20] and were compared against the controls. The subgroup of PH patients (20) with preserved LVEF included patients with an echocardiographic diagnosis of diastolic dysfunction (patients with heart failure with preserved ejection fraction (HFpEF)), and patients without evidence of systolic or diastolic dysfunction (Figure 1).

### 2.1. Cardiac MRI (cMRI)

cMRI was performed on a Siemens 1.5 T MRI (Siemens, Erlangen, Germany) using phased-array cardiac coil. The following images were reviewed in the cMRI study in all subjects: balanced steady-state free precession (bSSFP) 2D cine imaging was obtained on the short-axis (base to apex), and in the four-chamber, two-chamber, and left ventricular outflow tract views followed by 2D single shot bSSFP, and segmented gradient recalled echo (GRE) delayed late gadolinium enhanced imaging. 

### 2.2. CMR Image Analysis

The de-identified cMRI images were analyzed by two readers with more than five years of experience in cardiac imaging in consensus (SP and PN) using an FDA approved freely available software dubbed Segment (version 3.0: http://segment.heiberg.se, accessed on 28 April 2021) [21]. This software was used for selecting the desired end-systolic mid-ventricular short-axis [22] image from bSSFP cine series for further texture analysis, as performed in prior cMRI studies [22]. 

### 2.3. Right Heart Catheterization

Electronic medical records of the selected patients in the PH group were retrieved, and information regarding right heart pressure including mean pulmonary artery pressure, pulmonary vascular resistance, and pulmonary capillary wedge pressure were recorded. 

### 2.4. Image Pre-Processing

The de-identified DICOM short-axis mid-ventricular slice was transferred to the texture software MaZda 4.6 [23]. Image normalization was performed to make sure that the features were reflective of only texture and were not affected by image contrast or overall brightness [10]. This was performed within the texture software by rescaling the histogram data, so the gray scale range was between image mean and three standard deviations (mean − 3 SD and mean + 3 SD).

### 2.5. Image Segmentation

Segmentation was performed on a mid-ventricular end-systolic slice [22] by SP and PN in consensus. A left ventricular myocardium mask was manually segmented using the pencil tool within MaZda software. Care was taken to include only the myocardium and avoid papillary muscles and the blood pool (Figure S1). 

### 2.6. Texture Features Extraction

For each mask, 348 features were extracted using the MaZda software. These included histogram (9), co-occurrence matrix (220), run-length matrix (20), gradient (5), autoregressive (5), geometrical (73), and wavelet (16) features. Details about these features are provided elsewhere [24,25].

### 2.7. Feature Selection

Feature selection was a critical piece of the model building process due to the large size of the feature set relative to the sample size. Feature reduction was performed to exclude irrelevant, redundant, duplicated, highly correlated features and to reduce data dimensionality and complexity. This was performed by using three feature selection methods: a linear combinations filter (lincomb), a high correlation filter (corr), and a principal components analysis (PCA). The linear combinations filter addressed both collinearity and dimension reduction by finding linear combinations of two or more features and removing columns until the feature set was full rank. The high correlation filter removed variables from the feature set which had a large absolute correlation. The number of components retained in the PCA transformation was determined by specifying the fraction of the total variance that should be covered by the components. The threshold for the largest allowable absolute correlation for the high correlation filter was set to 0.6, and for the fraction of total variance in the PCA transformation was set to 0.9. These thresholds were chosen to retain as much information as possible while providing enough dimension reduction to allow model fitting. Prior to any feature selection, all variables were standardized. These feature selection methods were implemented using the recipes package [26] in software R version 4.0.2 (R Foundation for Statistical Computing, Vienna, Austria). 

### 2.8. Model Fitting

Twelve different predictive models were fit to determine the best classifier and feature selection combination for each feature set. These models were chosen to encompass a variety of classifiers including linear, non-linear, and ensemble classifiers. The linear classifiers evaluated were linear, logistic, ridge, elastic net, and LASSO (least absolute shrinkage and selection operator) regression. The non-linear classifiers evaluated were single-hidden-layer feedforward neural network, support vector machine (SVM) with a polynomial kernel, and SVM with a radial kernel and multilayer perceptron (MLP). Finally, the ensemble classifiers evaluated were random forest, generalized boosted regression model (GBRM), and boosting of classification trees with AdaBoost.

### 2.9. Model Performance Evaluation

**Figure 2:**
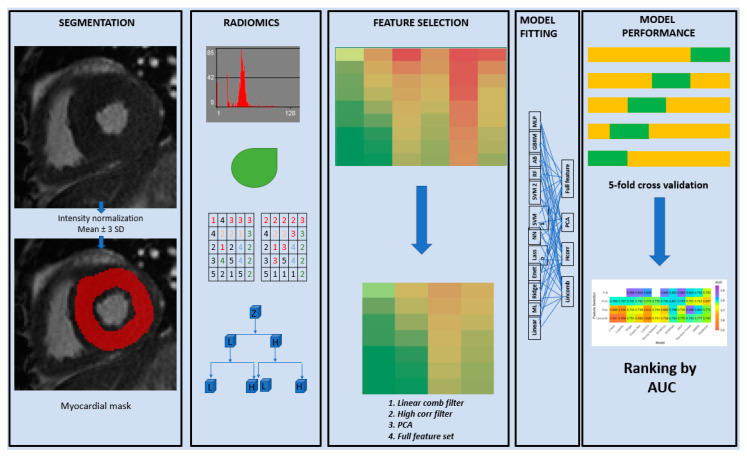
Radiomics workflow. Overall workflow of the entire process from segmentation to texture feature selection and model validation.

Each model was fit using the three aforementioned feature selection techniques as well as the entire feature set (full) without any feature selection, except for the linear regression, logistic regression, and the neural network which cannot be fit with the full feature set. This is because the model parameters cannot be uniquely estimated in linear and logistic regression models when the number of features is much larger than the sample size. For neural networks however, the problem was more related to excessive computational requirements. The model fitting process was also repeated using only the PH subgroup compared to the normal control group. The overall workflow of the entire process from segmentation to texture feature selection and model validation is summarized in Figure 2.

## 3. Statistical Analysis 

Continuous data was reported as mean ± standard deviation or median (IQR), where appropriate. Group differences were tested using the two-sample *t*-test, Wilcoxon rank-sum test, or the chi-square test, and *p*-values below 0.05 indicated a significant difference between the two groups. 

Model fitting and cross-validated predictive performance was implemented using the MachineShop [27] and RSNNS [28] packages, in software R version 4.0.2 (R Foundation for Statistical Computing, Vienna, Austria) [29]. Five-fold repeated cross-validation with five repeats was performed to evaluate the predictive performance of each model. When necessary, tuning parameters were selected using nested cross-validation to avoid bias. Hyperparameter tuning was done over a grid of parameter values for the default model hyperparameters identified by the MachineShop software. For example, for the neural network model, the hyperparameters were tuned to define the number of units in the hidden layer and the weight decay value. The feature selection techniques were carried out within each cross-validated split of the data, so as not to bias the estimate of predictive performance. Predictive performance was measured with the area under the receiver operating characteristic curve (AUC) for interpretability. As models were formulated to predict PH, AUC estimated the probability that a randomly selected subject that had PH would have a greater predicted value than a randomly selected normal control. Higher AUC values indicated better predictive performance. The R code used to perform the analysis is provided in the supplementary file.

## 4. Results

### 4.1. Patient Characteristics

No significant differences were found between the two groups for age, body surface area (BSA), smoking status, associated diabetes mellitus, and hypertension except for BMI. Table 1 presents the demographics and cMRI characteristics available for both groups. 

The majority of patients with PH (26) were classified as WHO group 2. Table 2 displays summary statistics for additional variables (only available for the PH group).

### 4.2. Model Performance on Primary Analysis

**Figure 3:**
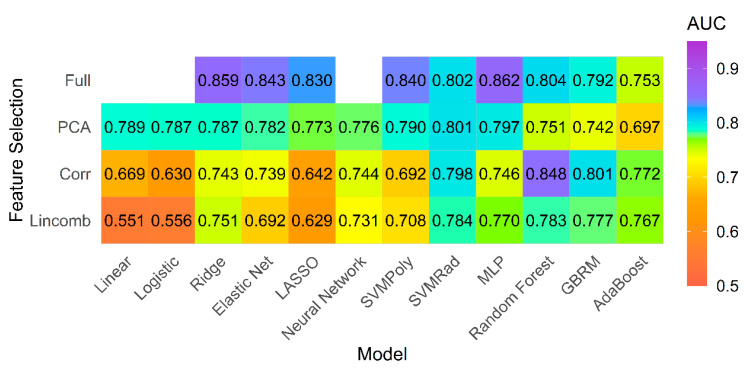
Model performance for primary analysis. Mean AUC for all models and feature selection combinations for primary analysis including all patients with pulmonary hypertension and controls.

The best model performance was seen for the multilayer perceptron (MLP) network model classifier using the full feature set with cross-validated AUC 0.862 and accuracy of 78% (Figure 3).

The summary statistics for the AUC of the top five models with the highest mean AUC is provided in Table 3.

### 4.3. Model Performance on Subgroup of PH Patients with Preserved Ejection Fraction (EF)

**Figure 4:**
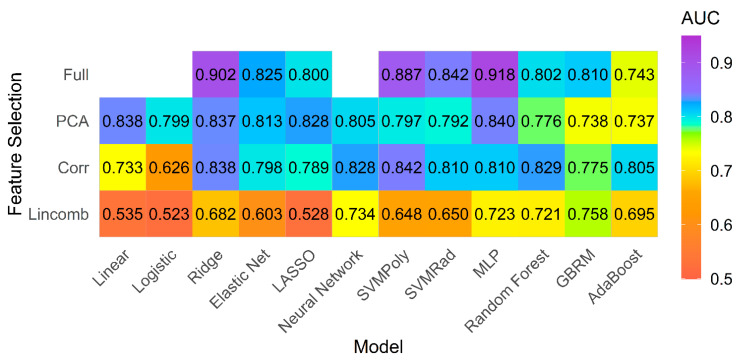
Model performance for subgroup analysis. Mean AUC for all models and feature selection combinations for subgroup analysis including patients with pulmonary hypertension and preserved left ventricle ejection fraction (>50%) and controls.

There were 20 patients in the PH group with LVEF ≥ 50% (15 patients had diastolic dysfunction on echocardiogram, and 5 patients had neither diastolic nor systolic dysfunction). The texture models performed well in comparison of normal subjects with this subgroup of PH patients with preserved LV ejection fraction. The best performing model was the MLP model fit using the full feature set, which achieved a cross-validated AUC of 0.918 and accuracy of 80% (Figure 4). 

Table 4 provides the summary statistics for the AUC of the top five models with the highest mean AUC using each feature set for the PH subgroup with preserved ejection fraction.

### 4.4. Overall Performance for Both Groups

Performance metrics for the best models in each feature set for both the groups are provided in Table 5. The top models were selected using the cross-validated AUC, where predictive performance was measured repeatedly on different held-out test sets which were not used in model fitting. In contrast, observed AUC was obtained by re-training the best models on the full data.

### 4.5. Feature Importance 

Feature importance was evaluated for the highest performing model for both primary and subgroup analysis (Tables S1 and S2). Since feature importance is not defined for MLP, we defined feature importance for the next best performing model (ridge regression). The top ten features were a combination of geometry, co-occurrence matrix, autoregressive, histogram, and wavelet features. These features were a combination of shape, first and higher order texture features. 

## 5. Discussion

This study uniquely demonstrates that radiomics features extracted from mid-left ventricular myocardium can non-invasively differentiate between patients with and without PH. 

Our pilot study demonstrates that the use of radiomics-based machine learning using texture features from cardiac MRI has excellent diagnostic performance for the differentiation between patients with and without PH. More importantly, our study demonstrates that the radiomics-based models perform well even for PH patients that have no systolic dysfunction. This is important information that may help non-invasive identification of PH patients prior to systolic cardiac decompensation. Multilayer perceptron (MLP) was the top performing classification model for both primary and subgroup analysis. 

We found the MLP model to be the best performing model. The MLP model is a type of feedforward artificial neural network that has an input layer, an output layer, and multiple hidden layers. MLP helps in distinguishing data that is not linearly separable [30]. The top five models for both primary and subgroup analysis were all fit to the full feature set, except for the fourth-best model which was fit using a high correlation filter. 

We evaluated forty-five different combinations of classifier models and feature selection strategies and found that model performance was variable for the best and the worst performing models (AUC 0.523–0.918). Classifier model performance depends both on the available data and on the classification task problem. Prior cMRI studies using radiomics performed in patients with myocarditis, hypertrophic, or other forms of cardiomyopathy or prognostication of tachyarrhythmias have only assessed single or limited machine learning models [16,17,18,24,31,32]. In our study, we explored the impact of multiple different models and feature selection techniques on myocardial radiomics performance. Our study provides evidence that the overall performance is based on the chosen combination of models and feature selection methods. As such, prediction based on a single model may be suboptimal. We conclude that further studies should assess multiple models and feature selection strategies to ensure a more rigorous model selection process.

Our results demonstrate similar performance for the PH subgroup with relatively preserved LVEF and provides evidence that radiomics features remain unaffected by the variations in ventricular ejection fraction. This is important, as by using radiomics we may be able to identify PH patients at risk of cardiac dysfunction early, while they still have normal ejection fraction. 

Feature reduction is often thought to be an essential part of the model building process, either to reduce computational time or to reduce noise in the data. In our study, we compared filtering methods (high correlation and linear combinations filter), feature extraction using PCA, embedded feature selection methods from penalized classifiers and tree-based ensemble models (ridge, elastic net, LASSO, random forest, GBRM, adaBoost), and models fit using all features. The embedded feature selection performed by the penalized classifiers and tree-based models selected feature subsets during the process of selecting the optimal model. In contrast, filter methods performed feature selections that were independent of the classifier performance. Our primary goal was assessing predictive performance, and we found that MLP models fit using all available features performed best for both the primary and subgroup analyses. While the MLP model does not perform embedded feature selection as each feature has an associated non-zero weight in the input layer, its ability to model non-linear relationships in all features is found to be advantageous in this setting.

Our results stress that model performance is dependent on the type of classifier and feature selection method chosen, as well as the dataset that the model is applied to. For example, in the primary analysis the penalized classifiers showed improved performance when fit to the full feature set (AUC 0.830–0.859) compared to fitting with feature selection methods (AUC 0.629–0.787). On the other hand, the ensemble classifiers (random forest, GBRM, adaBoost), which also perform embedded feature selection, performed best in combination with the high correlation filter (AUC 0.772–0.848) compared to when the full feature set was used (AUC 0.753–0.804). Thus, the belief that a priori feature reduction is mandatory in model building may not always be true. 

Feature importance evaluation showed that multiple feature types were seen in the best performing models and belonged to different texture types. These findings suggest that different features may carry different textural information and thus inclusion of multiple feature types may improve the model performance. 

Besides limitations of retrospective data, we did not perform external validation to improve generalizability of the optimal model, i.e., the model with the best cross-validated performance fit to the full dataset. However, we performed fivefold cross-validation with five repeats to avoid overfitting and bias, and to validate our models. Additionally, we provided the optimal models using the entire studied group and the subgroup and R code to make predictions from these models in a supplementary file so they may be applied to new data in the future. For new data we would suggest fitting the model which had the best cross-validated mean AUC to the full dataset and then applying the re-trained model to new data.

Additionally, we did not evaluate radiomics from right ventricular myocardium. The impact of PH is known to cause right heart strain and the effect of right ventricle myocardium texture on the models’ performance needs to be tested. In this study, we avoided the right ventricle myocardium because of its relatively thin wall compared with the left ventricle. Since the majority of PH cases in our study had WHO group 2 (PH secondary to left ventricle dysfunction), the association between radiomic features from LV myocardium and PH is plausible. However, our results may not be directly applicable to primary pulmonary hypertension and further studies with larger numbers of patients from a different WHO group PH will be needed to explore if there is any association with primary PH. Currently there are no known correlates of radiomic features with myocardial physiology, and that makes it difficult to understand the biological correlation of these texture features. To account for heterogeneity from other variables, the PH group and the control groups were age and gender-matched. The comparison groups were also matched for body surface area (BSA), smoking status, diabetes mellitus, and hypertension. However, the PH group had significantly higher body mass index (BMI). This is due to the retrospective nature of the study and higher incidence of PH among obese individuals. This difference could affect the model performance, and future studies should be designed to account for potential confounding variables. 

Besides PH, cardiac MRI-derived radiomic features may help in differentiating patients with left ventricular hypertrophy based on etiology (athlete’s heart, hypertensive heart disease, amyloidosis, sarcoidosis, or hypertrophic cardiomyopathy) and assessment of myocardial infarction and viability. Further studies should explore semiautomatic or fully automatic segmentation of myocardium and extraction of radiomic features to minimize manual input and time. In addition, evaluation of radiomic features from an entire cardiac cycle may be performed.

## 6. Conclusions

Cardiac MRI-based texture feature extraction using radiomics and machine learning demonstrated excellent performance in identifying patients with PH both before and after the onset of LV systolic dysfunction. The MLP model fit using a full feature set was the best performing model for the entire population group and PH subgroup with preserved LVEF. The performance of machine learning models varies based upon the chosen feature selection and model combinations.

## Figures and Tables

**Table 1:** Demographics, co-morbidities, and cardiac MRI features of control and pulmonary hypertension groups.

	Normal (*n* = 30)	Pulmonary Hypertension (*n* = 42)	*p* Value
Age ^a^	49.53 ± 12.72	54.45 ± 17.42	0.1706
Number of Women (%)	16 (53.3)	23 (54.8)	0.9045
BMI ^a^	28.82 ± 6.51	34.63 ± 9.00	0.0022
BSA ^a^	1.96 ± 0.35	2.08 ± 0.27	0.1490
RVEF ^b^	55.50 (53.00–61.00)	39.50 (29.0–47.75)	<0.0001
LVEF ^b^	62.00 (58.00–67.00)	45.50 (21.0–57.83)	<0.0001
RVEDVI ^b^	72.24 (62.07–82.63)	97.85 (73.99–120.71)	0.0003
LVEDVI ^b^	76.73 (65.06–86.09)	95.21 (67.45–144.70)	0.0511
Smoking Status—*n* (%)			0.1444
Current	2 (6.67)	3 (7.14)	
Former	7 (23.33)	19 (45.24)	
Never	21 (70.00)	20 (47.62)	
DM—*n* (%)			0.0663
No	26 (86.67)	26 (61.90)	
Yes	4 (13.33)	15 (38.10)	
Number with Hypertension (%)	14 (46.67)	25 (59.52)	0.2804

^a^ mean +/− sd. ^b^ median w IQR in (). BMI: body mass index; BSA: body surface area; RVEF: right ventricle ejection fraction; LVEF: left ventricle ejection fraction; RVEDVI: right ventricle end-diastolic volume indexed; LVEDVI: left ventricle end-diastolic volume indexed; DM: diabetes mellitus.

**Table 2:** Right heart catheterization (RHC) characteristics of the pulmonary hypertension (PH) group with NYHA classification and World Health Organization PH class distribution.

Parameters	Pulmonary Hypertension (PH) (*n* = 42)
PA Pressure ^a^	37.00 (22–60)
PVR ^a^	2.25 (0.91–9.95)
PCW ^a^	22.00 (9–35)
Dur b/*n* RHC and Cardiac MRI (days)	6.00 (0–30)
WHO Class—*n* (%)	
1	3 (7)
2	26 (62)
3	1 (2.4)
1 & 2	1 (2.4)
1, 2 & 3	1 (2.4)
2 & 3	9 (21.4)
5	1 (2.4)
NYHA Class—*n* (%)	
1	2 (4.76)
2	5 (11.90)
3	23 (54.76)
4	6 (14.29)
No	2 (4.76)
Not Available	4 (9.52)

^a^ medians w range (min–max) in (). PA: pulmonary artery; PVR: pulmonary vascular resistance; PCW: pulmonary capillary wedge pressure; RHC: right heart catheterization; NYHA: New York Heart Association Classification; WHO: World Health Organization.

**Table 3:** Top five models selected to fit for entire group (all PH versus control).

Model	Feature Selection	Mean	SD	Median	Min	Max
MLP	full	0.862	0.066	0.852	0.759	0.862
Ridge	full	0.859	0.063	0.852	0.750	0.859
RF	corr	0.848	0.081	0.854	0.630	0.848
Enet	full	0.843	0.094	0.854	0.667	0.843
SVM Poly	full	0.840	0.078	0.852	0.685	0.840

MLP: multilayer perceptron; RF: random forest; Enet: elastic net; SVM Poly: support vector machine with a polynomial kernel; corr: high correlation filter.

**Table 4:** Top five models selected to fit for PH subgroup (PH subjects with preserved ejection fraction versus controls).

Model	Feature Selection	Mean	SD	Median	Min	Max
MLP	full	0.918	0.089	0.917	0.708	1.000
Ridge	full	0.902	0.129	0.958	0.542	1.000
SVM Poly	full	0.887	0.152	0.958	0.417	1.000
SVM Poly	corr	0.842	0.164	0.875	0.417	1.000
SVM Rad	full	0.842	0.155	0.875	0.417	1.000

MLP: multilayer perceptron; SVM Poly: support vector machine with a polynomial kernel; SVM Rad: support vector machine with a radial kernel; corr: high correlation filter.

**Table 5:** Performance metrics for best performing texture models on each feature set for the entire studied group (PH vs. controls) and subgroup with preserved ejection fraction.

Feature Set	Model	Feature Selection	Observed AUC	CV AUC	CV Accuracy	CV Sensitivity	CV Specificity
LV mask (entire group)	MLP	full	0.998	0.862	0.783	0.794	0.767
LV mask (PH subgroup)	MLP	full	1.000	0.918	0.808	0.740	0.853

MLP: multilayer perceptron; full: full feature set; AUC: area under the curve; Observed AUC: AUC when final selected model is fit to full dataset; CV: cross-validated.

## Data Availability

The .rds files of the best trained models for the entire group analysis (MLP model) and the subgroup analysis (MLP) and R code to load the models and compute performance metrics on the observed data are provided as a supplementary file.

## Supplementary Materials

The following are available online at https://www.mdpi.com/article/10.3390/jcm10091921/s1. Figure S1. Myocardial mask segmentation. A single mid-ventricular short-axis slice from balanced 2D steady-state free precision cine cardiac MRI shows generation of left ventricular myocardial mask (red). Table S1. Feature importance for texture variables for the whole group for the best performing model (random forest with full feature set). Table S2. Feature importance for texture variables for pulmonary hypertension subgroup for the best performing model (random forest with high correlation filter). R code for models.
