# Supplementary material for: Radiomics Detection of Pulmonary Hypertension via Texture-Based Assessments of Cardiac MRI: A Machine-Learning Model Comparison—Cardiac MRI Radiomics in Pulmonary Hypertension
Source: J Clin Med. 2021 Apr 28;10(9):1921. doi: 10.3390/jcm10091921 (PMC8125238; doi:10.3390/jcm10091921)
Supplement: Supplementary file 1 [file jcm-10-01921-s001.zip › SUPPLEMENTARY MATERIAL-PROOF READ.pdf]

Journal of
Clinical Medicine

SUPPLEMENTAL MATERIAL

Radiomics Detection of Pulmonary Hypertension via Texture-Based Assessments of Cardiac MRI: A Machine-Learning Model Comparison—Cardiac MRI Radiomics in Pulmonary Hypertension

Figure S1. Myocardial mask segmentation. A single mid ventricular short-axis slice from balanced 2-D steady state free precision cine cardiac MRI shows generation of left ventricular myocardial mask (red).

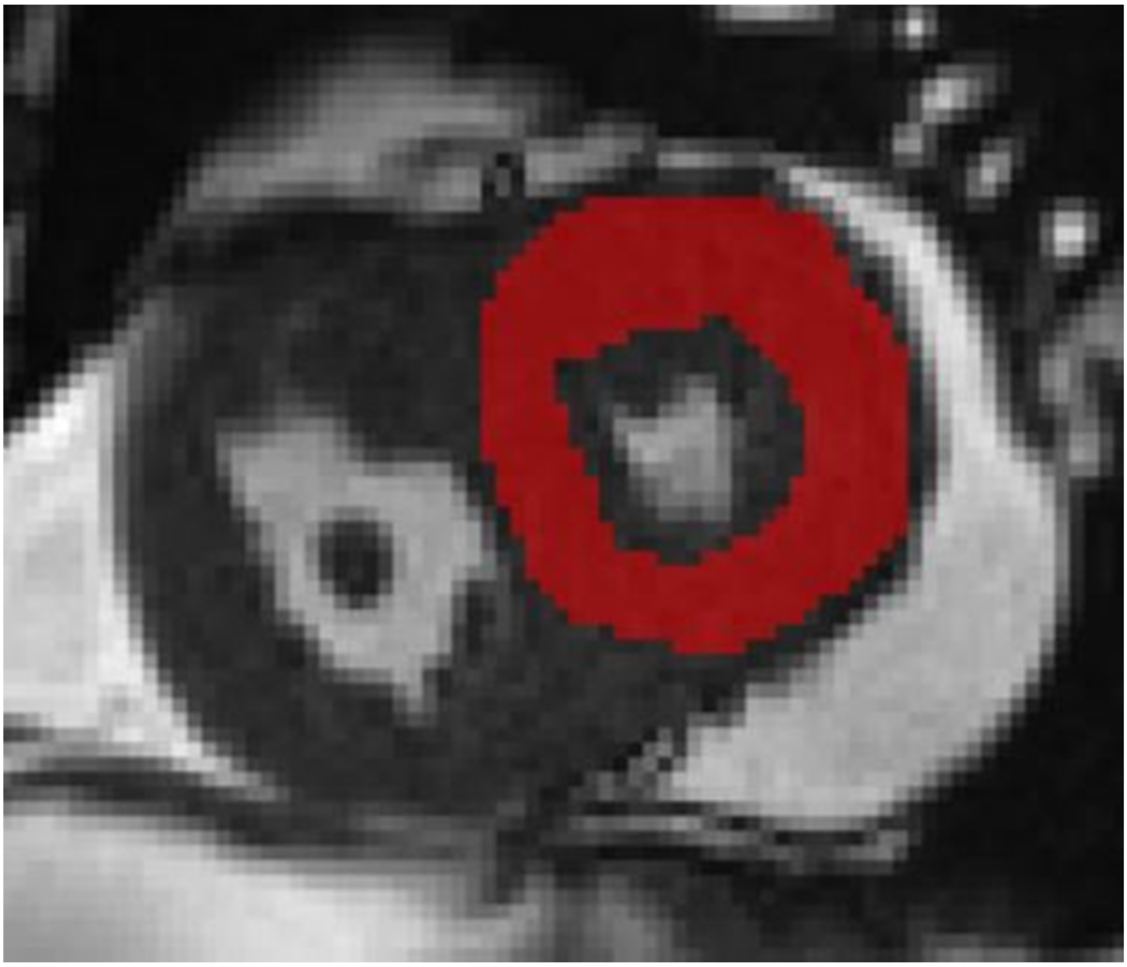

Table S1. Feature importance for texture variables for whole group for best performing model where feature importance is defined (Ridge with full feature set).

Feature	Importance
eslv_GeoRf	100
eslv_GeoLsz	38.24698877
eslv_GeoW5b	32.81326741
eslv_S_2_2_Correlat	30.20943885
eslv_WavEnLH_s_4	29.42206805
eslv_GeoM2y	22.6401386
eslv_Perc.01.	22.21651047

eslv_GeoW7	18.06157753
eslv_S_3_3_Correlat	17.43654819
eslv_WavEnHH_s_2	16.89151075

Eslv- End-systolic LV mask.

Table S2. Feature importance for texture variables for pulmonary hypertension sub-group for best performing model where feature importance is defined (Ridge with full feature set).

**Feature**	**Importance**
eslv_WavEnHH_s_1	100.000
eslv_WavEnHH_s_2	92.573
eslv_WavEnHL_s_4	86.476
eslv_GeoRf	84.545
eslv_WavEnHH_s_3	82.088
eslv_Teta3	68.262
eslv_GeoS2	67.557
eslv_GeoW10	66.680
eslv_Kurtosis	63.359
eslv_S_0_5_DifVarnc	61.528

Eslv- End-systolic LV mask.
